# Vitamin C in Oral Lichen Planus: Pathobiological Rationale and Therapeutic Potential

**DOI:** 10.3390/ijms262411887

**Published:** 2025-12-09

**Authors:** Małgorzata Mazurek-Mochol, Martyna Mochol, Agnieszka Chamarczuk, Mariusz Lipski

**Affiliations:** 1 Department of Periodontology, Pomeranian Medical University in Szczecin, Powstańców Wlkp 72, 70-111 Szczecin, Poland; 2 Department of Preclinical Conservative Dentistry and Preclinical Endodontics, Pomeranian Medical University in Szczecin, Powstańców Wlkp 72, 70-111 Szczecin, Poland

**Keywords:** oral lichen planus, vitamin C, ascorbic acid, oxidative stress, antioxidant therapy, immune modulation, epithelial integrity, inflammation, adjunctive treatment

## Abstract

Oral lichen planus (OLP) is a chronic immune-mediated inflammatory disorder of the oral mucosa associated with oxidative stress, epithelial degeneration, and persistent T-cell–driven inflammation. Despite numerous therapeutic approaches, disease control and mucosal healing remain challenging. This review aims to summarize current evidence on the biological functions of vitamin C (VitC) relevant to OLP pathogenesis and to evaluate its potential as an adjunctive therapeutic agent. A comprehensive literature review was performed to identify studies exploring the molecular mechanisms of VitC in oxidative stress modulation, epithelial integrity, and immune regulation, as well as clinical and experimental data assessing its therapeutic outcomes in OLP. VitC has various effects relevant to OLP, including scavenging of reactive oxygen species, stabilization of epithelial structures, regulation of apoptosis, and modulation of cytokine expression. Preliminary studies indicate beneficial effects on oxidative balance, inflammation, and tissue repair; however, the evidence is still limited and varies among different study designs and formulations. VitC represents a biologically plausible and safe adjunct in OLP management, acting at multiple pathobiological levels. Further well-designed clinical trials are needed to establish optimal dosing, delivery strategies, and long-term outcomes.

## 1. Introduction

Oral lichen planus (OLP) is an immune-mediated inflammatory disease of the oral mucosa, chronic and affecting about 1–2% of the world population, predominantly in females, and most commonly in middle age [1,2]. It presents itself clinically in reticular, atrophic, erosive, papular, plaque-like, or bullous, mostly affecting the buccal mucosa, tongue, and gingiva [3,4]. The oral functioning and quality of life can be severely affected by pain, burning, and greater sensitivity to spicy or acidic foods. Histologically, OLP is typified by a band-like subepithelial lymphocytic infiltrate that is predominantly composed of CD8+ cytotoxic T cells, basal keratinocyte degeneration, and destabilization of the basement membrane [5].

Topical corticosteroids remain the first-line treatment for symptomatic or erosive OLP; however, several other therapeutic approaches have also been shown to be effective, including calcineurin inhibitors, retinoids, photodynamic therapy (PDT), or systemic immunomodulators [6,7]. Despite symptomatic relief in many patients, frequent relapses, mucosal thinning, and the chronic-relapsing nature of OLP necessitate long-term management. The adverse effects of steroids, including mucosal candidiasis or xerostomia, highlight the necessity of adjunctive or alternative methods that might focus on the underlying oxidative-inflammatory pathology of the disease, but not just its symptoms [7].

Oxidative stress, described as a state in which an imbalance occurs between the production of reactive oxygen and/or nitrogen species (ROS/RNS) and the capacity of the biological system to detoxify these reactive intermediates, or to repair the resulting damage [8], is becoming an increasingly important mediator of the immune-mediated damage in OLP [9,10]. High concentrations of lipid-peroxidation products (salivary malondialdehyde (MDA), 8-isoprostanes) and oxidized nucleic-acid adducts (8-hydroxy-2′-deoxyguanosine) are also consistently observed in serum and saliva of patients with OLP, and there is a reduction in antioxidant defences (glutathione, superoxide dismutase, catalase, total antioxidant capacity) [10,11]. NF-kB and MAPK are activated by ROS to induce the expression of pro-inflammatory cytokines (TNF-α, IL-6, IL-17) and inhibit epithelial regeneration [4]. This redox imbalance offers a biologically plausible target to antioxidant modulation as an OLP therapy [12].

Vitamin C (VitC) is a major water-soluble antioxidant and enzymatic cofactor in collagen hydroxylation, epithelial barrier preservation, and immune regulation [13]. It removes ROS and RNS, restores oxidized vitamin E, and suppresses NF-kB-mediated cytokine transcription [14]. VitC improves the activity of neutrophils and macrophages and suppresses the overproduction of Th1/Th17 in inflammatory mucosal conditions, thus facilitating inflammatory resolution. Since OLP is a combination of long-term oxidative damage, epithelial degeneration, and slow wound healing, the biochemical profile of ascorbate is consistent with the pathophysiology of the disease. It has been observed that salivary VitC is lower, and the total antioxidant capacity is lower in OLP, which is indicative of greater local consumption during oxidative stress [9,10,15]. In addition, VitC deficiency or sub-optimal condition has been associated with delayed mucosal healing and increased inflammatory signalling in associated oral diseases [16].

This review is a critical analysis of the interaction between VitC and OLP, which combines mechanistic, biochemical, and clinical views. It will discuss the way VitC can modulate disease mechanisms, including oxidative stress, epithelial damage, and immune dysregulation, and assess the current evidence on its biomarker and therapeutic potential. Practical considerations of VitC use, such as routes of delivery, safety, and aspects that can alter patient response, are also discussed. Combining the results of experimental, biomarker, and clinical research, this review identifies the existing strengths and limitations of the evidence base and provides major directions for further research in the field of antioxidant modulation in OLP.

## 2. Pathobiology of Oral Lichen Planus and Potential Sites of Vitamin C Action

### 2.1. Immunopathology of OLP

On histopathological examination, OLP is characterized by a dense, band-like infiltrate of T lymphocytes in the superficial lamina propria, which is closely adjacent to the basal epithelial layer, and apoptotic degeneration of basal keratinocytes. The central effectors are the CD8+ cytotoxic T cells, which cause the death of keratinocytes by Fas-FasL interactions, TNF-α, and exocytosis of perforin and granzyme B [1]. The subsets of helper T-cells, especially Th1 and Th17, increase local inflammation through the production of cytokines, including IFN-g, TNF-α, and IL-17, which perpetuate epithelial injury [3]. The breakdown of the basement-membrane zone, facilitated by matrix metalloproteinases (in particular, MMP-9) and mast-cell proteases, allows further T-cell infiltration of the epithelium and the continuation of the cycle of tissue destruction [1,3,5].

### 2.2. Co-Driving of Oxidative Stress

In addition to immune dysregulation, oxidative stress is also becoming a significant cofactor in OLP. Some studies have found high levels of oxidative-damage products, including MDA, F2-isoprostanes, and 8-hydroxy-2′-deoxyguanosine (8-OHdG) in serum or saliva of OLP patients, along with impaired antioxidant defences, including low levels or activity of glutathione, catalase, superoxide dismutase, and total antioxidant capacity [3,9,11,17]. As an illustration, Singh et al. (2022) found that salivary MDA is significantly higher in OLP patients than in healthy controls [11], and Wang et al. (2021) established across several populations that there are increased oxidant markers and reduced antioxidant markers in OLP patients [9]. These redox imbalances are likely to aggravate mucosal damage by increasing the production of NF-kB-regulated cytokines, increasing the expression of MMPs, and disrupting epithelial-repair activities [4].

### 2.3. Biological Roles of Vitamin C in Relation to OLP

VitC has multiple levels of action that may affect OLP pathogenesis. As an antioxidant and redox signalling molecule, ascorbate neutralises ROS and RNS, restores oxidized antioxidants including vitamin E, and regulates redox-sensitive transcription factors such as NF-kB, which leads to reduced production of pro-inflammatory cytokines [13]. In terms of immune modulation, VitC helps to maintain a healthy balance in immune responses by increasing the activity of phagocytes and natural killer cells and by inhibiting the overproduction of cytokines; it can also be used to reestablish the balance between Th17 and T regulatory cells, as shown in models of autoimmune and inflammatory diseases [14]. With regard to epithelial and connective tissue repair, VitC is a vital cofactor for prolyl and lysyl hydroxylases, so it is required for collagen production and stabilization, and sufficient ascorbate maintains the integrity of basement membranes and facilitates epithelial repair, processes that are disrupted in OLP. Uptake and local protection of VitC depend on sodium-dependent VitC transporters (SVCT1 and SVCT2), with SVCT2 being highly expressed in cells with high metabolic activity or under inflammatory conditions, where it sustains intracellular antioxidant potential during oxidative stress. Finally, VitC may modulate matrix remodelling by indirectly inhibiting MMP-9 expression and activity through suppression of oxidative signalling and NF-kB activation. Because MMP-9 participates in the dismantling of basement membranes and the migration of immune cells in OLP, this represents a plausible defensive mechanism [5,13].

### 2.4. Integrative Mechanistic View

**Figure 1:**
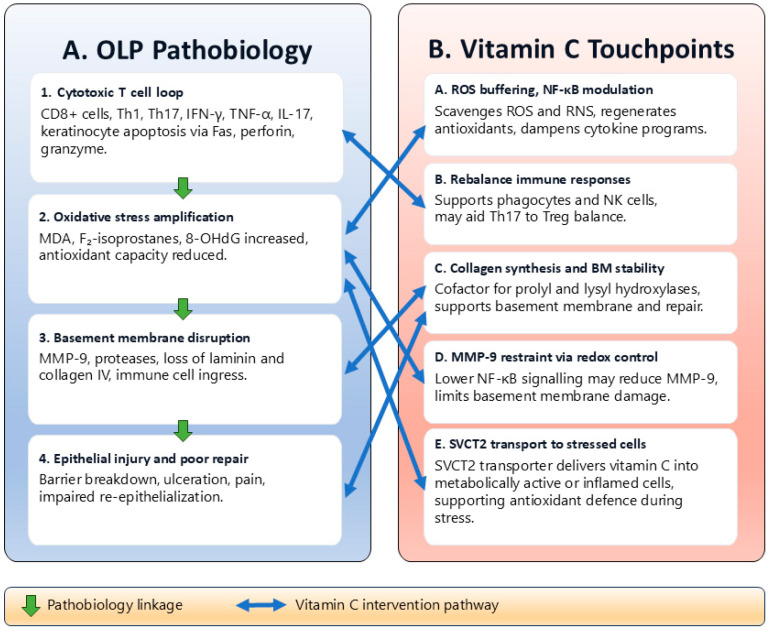
Pathobiology of oral lichen planus (OLP) and proposed vitamin C (VitC) touchpoints. VitC may counteract key pathogenic mechanisms in OLP by reducing oxidative stress, modulating immune responses, stabilizing the basement membrane, and promoting epithelial repair. References: 1. [1,3,5], 2. [4,9,11,17], 3. [1,3,5], 4. [1,3,4,5,9,11,17], A. [13,14], B. [14], C. [13], D. [5,13], E. [13].

Combined, VitC may inhibit multiple interdependent mechanisms in OLP, such as excessive oxidative stress, chronic cytokine synthesis, degradation of the basement membrane, and inadequate epithelial repair. This mechanistic framework is supported by the consistent finding of decreased salivary ascorbate and antioxidant capacity in OLP patients and indicates that oral tissues have a special vulnerability to VitC deficiency. Despite the limited number of direct clinical trials, there is convergent biochemical and immunologic evidence to consider additional investigation of VitC as an adjunctive treatment of OLP. Figure 1 summarizes these interrelated mechanisms and shows the key pathogenic pathways in OLP and the suggested location of VitC action.

## 3. Biomarker Evidence for Vitamin C in OLP

VitC is a key redox homeostat, epithelial, and inflammatory regulator, which directly overlaps the pathobiology of OLP. Since the disease is characterized by chronic inflammation of the mucous membrane and oxidative environment, several studies have examined VitC status in salivary, serum, and composite oxidative-stress environments. Overall, these studies suggest that VitC depletion and redox imbalance in OLP is a trend, but the evidence strength differs depending on the compartment and methodology.

### 3.1. Salivary Vitamin C

Saliva test is a convenient, non-invasive method of measuring local oxidative homeostasis. Most studies in OLP show that salivary ascorbate decreases significantly in comparison to healthy controls. Abdolsamadi et al. (2014) measured vitamins A, E, and C in saliva and found significantly low VitC levels and increased lipid-peroxidation products (MDA) in OLP (*p* < 0.001) [15]. Similar findings were made by Rai et al. (2008), who reported lower mean salivary VitC in OLP (0.68–0.79 mg/mL) compared to controls (1.01–1.11 mg/mL) and a positive correlation between VitC and E (r = 0.65, *p* < 0.05) [18]. These findings are supported by meta-analytic syntheses. Wang et al. (2021) showed a steady reduction in salivary VitC in pooled oxidative-stress studies [9]. Amirchaghmaghi et al. (2016) also reported a significant increase in the plasma level of 8-isoprostane in patients with OLP compared with the control group [19]. The evidence base is still weak, but its directional consistency is remarkable.

Salivary redox research in OLP focuses on enzymatic and composite indicators, such as superoxide dismutase (SOD), glutathione peroxidase (GPx), TAC, and MDA, and not on the quantification of micronutrients. Shirzad et al. (2014) demonstrated increased MDA and decreased TAC in erosive OLP (*p* < 0.0001) [20], Darczuk et al. (2016) less total glutathione (GSH) and TAC with more thiobarbituric acid reactive substances (TBARS) [17], and Rekha et al. (2017) more SOD and MDA with less GPx [21]. Shirzaiy et al. (2022) reported the reduction in salivary SOD, GPx, and albumin [22]. Together, these data support a locally oxidizing environment that is likely to be the cause of ascorbate consumption.

The decrease in salivary VitC can be due to augmented local oxidative consumption and not a deficiency in the body. Between-study heterogeneity is added by the variability (stimulated vs. unstimulated samples, diurnal timing, stabilization). However, convergent evidence suggests that ascorbate loss is correlated with increased lipid peroxidation, which places salivary VitC as a close biomarker of mucosal oxidative stress.

### 3.2. Serum Vitamin C

Findings of systemic VitC are more heterogeneous. Tsunoda et al. (2022) found that in a Japanese cohort, untreated OLP patients had significantly lower serum VitC levels compared to controls (5.73 ± 2.34 vs. 9.22 ± 3.65 μg/mL; *p* = 0.0125) [23]. On the other hand, Rezazadeh and Haghighat (2021) did not identify any significant group difference in vitamins A, C, and E between OLP cases and controls, but mean values were lower in OLP [24]. Such discrepancies are probably preanalytical variations, small sample sizes, and non-dietary or fasting control.

Greater oxidative-stress data provide context; Wang et al. (2021) and Hatami et al. (2022) both reported reduced serum/plasma TAC and elevated MDA, which is a systemic redox imbalance towards oxidation [9,10]. These results, combined with others, suggest that higher VitC use in OLP is biologically plausible, even with varying absolute serum concentrations.

The interpretation of the differences in serum VitC can probably be based on the load of inflammatory processes and the intensity of oxidative stress. The rapid turnover of ascorbate in the presence of oxidative stress can lead to temporary systemic depletion, which is not well reflected in standard assays. Standardization of fasting status, preanalytic stabilization, and quantification (HPLC/LC-MS) should be performed in future studies to demystify systemic trends.

### 3.3. Oxidative Stress Panels and Vitamin C Correlations

Extensive oxidative-stress testing is always characterized by a general imbalance towards oxidation in OLP. Both saliva and serum show increased MDA and reduced TAC and SOD [9,15,20]. Upadhyay et al. (2010) established an increased MDA and reduced total antioxidant levels (TAL) in the serum of patients with OLP than in controls (*p* < 0.001) [25].

Further correlation studies between VitC depletion and oxidative injury are directly correlated. Abdolsamadi et al. (2014) demonstrated that salivary VitC and MDA had an inverse correlation in patients with OLP than healthy control group [15]. Tsunoda et al. (2022) showed lower serum VitC levels with lower levels of urinary oxidative stress marker 8-hydroxy2′-deoxyguanosine (8-OHdG) in patients with OLP compared to controls [23]. Such patterns highlight the role of ascorbate in the presence of oxidative stress, which is in line with its ability to act as a primary aqueous antioxidant and recycler of α-tocopherol (vitamin E).

Higher levels of MDA and 8-isoprostane are associated with erosive/atrophic forms of OLP [17,19,20,21,22], which are also more painful and cause more mucosal damage. Therefore, VitC depletion reflects the severity of diseases and can be used to stratify oxidative phenotypes. The addition of VitC with TAC, MDA, 8-isoprostane, SOD, and GSH may provide a strong composite biomarker panel to monitor the disease and also to evaluate the antioxidant treatment. Key studies measuring salivary and serum VitC, together with related oxidative-stress markers, are summarized in Table 1.

## 4. Clinical Evidence for Vitamin C as Therapy in OLP

There are no randomized controlled trials (RCTs) and prospective studies that have been conducted to test VitC alone in treating OLP. Descriptions of scorbutic oral lesions healing with ascorbic acid have been described in history and are not pathophysiologically similar to the OLP diagnostic criteria. Therefore, VitC monotherapy in OLP has no evidence to support it, and any possible advantage is theoretical, awaiting controlled studies.

The RCTs are the strongest evidence in which VitC was incorporated into antioxidant complexes. Belal et al. [26] conducted an RCT of 30 adults with erosive-ulcerative OLP and randomly assigned the participants to: (i) topical corticosteroid, (ii) topical corticosteroid and antifungal, or (iii) selenium-ACE (selenium and vitamins A, C, E) combined with topical corticosteroids plus antifungal. Six weeks later, the Selenium-ACE group experienced a significant reduction in pain and lesion reduction compared to the other groups. The authors concluded that selenium-ACE can be helpful in improving symptom management as a complement to conventional treatment.

In a 22-patient, double-blind, randomized controlled trial of desquamative gingivitis, 18 of whom had OLP, patients applied a bioadhesive gel of propolis + nanovitamin C + nanovitamin E or a placebo gel for four weeks. Both groups also showed improvements in clinical conditions, and there were no statistically significant differences between the groups. The test group had improved pain and oral-health-related quality of life (OHIP-14) scores, and no adverse effects were reported [27]. These data are in favour of the safety and local tolerability of VitC/E preparations in mucosal inflammatory lesions.

A meta-analysis by Bao et al. (2022) [28] combined 17 RCTs (*n* = 704) evaluating systemic and topical antioxidants in OLP, such as vitamin E, lycopene, curcumin, selenium-ACE, and others and discovered that there was a significant overall pain and lesion severity reduction with no adverse events. These findings support the importance of antioxidant therapy as a useful addition to corticosteroids.

Individual antioxidant trials not involving VitC, such as lycopene vs. placebo in resistant erosive and atrophic OLP [29,30], also demonstrated clinical and biochemical improvements, reinforcing the concept that redox modulation can alleviate OLP pathology.

The aggregate clinical data show that antioxidant treatment can alleviate the level of pain and the severity of lesions in OLP patients. Among the different combinations of antioxidants tested, those that contain VitC, including the Selenium-ACE regimen, seem to offer some quantifiable added effect when applied in combination with topical corticosteroid and antifungal therapy [26]. The fact that pain and mucosal healing have improved supports the hypothesis that the oxidative imbalance may be corrected to reduce the inflammatory process underlying OLP.

It should be noted, though, that there are no randomized controlled studies that have examined VitC as a monotherapy in OLP. The data available are all based on combination regimens or larger antioxidant studies, and as such, it is impossible to determine the independent effect of ascorbate. Although these studies are all pointing to the fact that VitC may positively influence redox homeostasis and tissue repair, more specific trials are needed to clarify the exact therapeutic role of VitC.

Nanovitamin gels and other topical preparations of VitC and E have shown excellent safety and local tolerability in patients with desquamative gingivitis and lesions of OLP [27]. Even though the small sample under study did not demonstrate any statistical superiority over placebo, the lack of adverse effects and the improvement in subjective outcomes (pain reduction and betterment of oral-health-related quality of life) suggest that such topical antioxidant preparations should be investigated further in larger and OLP-specific randomized trials.

Further studies are required to isolate the effects of VitC by comparing known doses of ascorbate alone and with other antioxidants using standardized clinical measures (Visual Analogue Scales (VAS), Thongprasom’s score, reticulation/keratosis, erythema, and ulceration (REU scores) and objective oxidative biomarkers (MDA, 8-isoprostane, TAC). These studies would help to understand whether VitC has a specific therapeutic effect or is more of a supportive cofactor in the context of more comprehensive antioxidant approaches to OLP management.

## 5. Formulations, Dosing, and Delivery of Vitamin C in OLP

VitC (ascorbate) exhibits non-linear, saturable intestinal absorption and tight renal regulation, which limit achievable plasma concentrations with oral dosing. Plasma saturation occurs at total intakes of ~200–400 mg/day, while higher oral doses yield only modest additional increases; much higher peaks are achievable only via intravenous administration. Because OLP involves epithelial inflammation, oxidative stress, and impaired wound healing, two delivery rationales exist: (i) systemic oral dosing to restore or maintain physiologic tissue levels and (ii) local intra-oral/topical delivery to target lesions directly while limiting systemic exposure [15,27,31,32,33,34].

No standardized OLP-specific monotherapy trials exist, so dosing should be viewed as adjunctive and status-repleting. A pragmatic range of 200–400 mg/day maintains near-saturation plasma levels and supports antioxidant status without exceeding gastrointestinal tolerance [31,32,33]. This dosage range parallels that used in antioxidant-combination studies showing modest symptomatic benefit in OLP [30]. In patients with documented or suspected hypovitaminosis C-suggested by reduced salivary or plasma levels—systemic repletion is particularly rational [9,15,24,35].

Because OLP lesions are mucosal and often erosive, local delivery can provide high lesion-surface exposure with reduced systemic dosing. Formulation design should ensure (i) chemical stability, (ii) physiological pH (≈6.5–7.0), (iii) mucoadhesion to prolong residence time, and (iv) comfort and non-irritancy.

Suitable vehicles include mucoadhesive gels, bilayer films, and lozenges. Sodium ascorbate or buffered L-ascorbic acid are preferred actives; hyaluronan- or chitosan-based matrices maintain hydration and adherence [16,34,36,37]. In vitro studies show concentration-dependent effects of ascorbate rinses on gingival fibroblast migration and wound closure, supporting topical rationale while emphasizing optimization of pH and exposure [38]. In adjunctive practice, patients should first be advised to maintain gentle oral hygiene and to avoid containing alcohol or highly acidic mouthwashes, which can aggravate OLP lesions [39].

Next-generation mucoadhesive systems, such as catechol-modified polymers and nanoparticle-enhanced films, provide stronger adhesion and sustained release for oral actives. These could stabilize and localize ascorbate in OLP, though direct data are lacking [16,34,37].

Key evidence gaps remain in several areas. There is a need to define the dose-response relationship for clinically used oral ranges of VitC supplementation, typically between 200 and 1000 mg per day, in relation to changes in clinical and molecular biomarkers, since current ranges are largely extrapolated from general vitamin C literature rather than derived from OLP-specific trials [15,27,31,32,33]. Further work is required to optimize topical vehicles and concentrations suitable for use on oral mucosa [16,34,36,37,38]. Controlled trials should evaluate combinations of VitC with topical steroids compared with steroid monotherapy [6,27]. Finally, future studies should stratify patients by OLP subtype and by the presence of comorbid conditions associated with increased oxidative stress [9,15,24,35].

## 6. Safety, Tolerability, and Drug Interactions

VitC has a generally good safety profile, particularly when used in physiological or moderate supplemental levels. Recent clinical and meta-analytic data indicate its high tolerability in a variety of disease conditions, including systemic inflammatory and infectious disease conditions [27,40,41,42,43]. High-dose VitC infusion in non-critical patients admitted to the hospital showed few adverse events and no rise in renal complications or electrolyte imbalances [43]. Likewise, there are low rates of gastrointestinal effects with oral VitC supplementation, mostly transient diarrhea, nausea, or abdominal cramping, which usually occur at doses of more than 1–2 g per day [40,42].

There is still a need to be cautious in patients with renal failure, history of nephrolithiasis, or glucose-6-phosphate dehydrogenase (G6PD) deficiency, in which excess ascorbate may theoretically trigger oxalate nephropathy or hemolysis [42,43]. VitC can have transient pro-oxidant action at pharmacologic levels, although no clinically significant toxicity has been proven in recent human trials [41,44].

There is a dearth of direct safety data in OLP, yet results of antioxidant intervention studies suggest an overall benign profile. A systematic review of antioxidant therapy in OLP (one of which involved VitC as a component of a combination regimen) reported consistent symptom improvement and no severe adverse events in the studies [27]. Topical formulations, especially those containing unbuffered L-ascorbic acid or acidic gels, can cause temporary stinging or burning of ulcerated mucosa, whereas buffered sodium ascorbate or vehicles based on hyaluronic acid can decrease the risk of irritation [9,27].

A meta-analysis of oxidative stress and antioxidant biomarkers in OLP in 2021 confirmed the presence of a significant redox imbalance (reduced total antioxidant capacity, increased malondialdehyde), which justified the use of antioxidant supplementation and not the safety issues [9,10]. Taken together, the existing data indicate that it has good tolerability and can be redox restored with no significant systemic toxicity.

The recent experience with high-dose VitC in other systemic conditions also contributes to its safety margin. Oral and intravenous VitC decreased mortality or enhanced physiological parameters in COVID-19 and sepsis cohorts without any significant adverse effects [42,43,45,46]. A pilot study of pharmacokinetics with single mega-doses of sodium ascorbate showed that hemodynamics were stable and there were no renal or metabolic complications, even at supraphysiological plasma concentrations [44]. These findings support the high tolerability of VitC when used in controlled conditions and indirectly support the potential of VitC in chronic inflammatory oral disease like OLP.

VitC has a low interaction potential with conventional OLP therapies. Topical corticosteroids, calcineurin inhibitors, or systemic retinoids have not been reported to have any significant pharmacodynamic or pharmacokinetic interactions [27,41]. Indeed, VitC can be used to supplement mucosal repair in corticosteroid therapy by promoting collagen synthesis and limiting oxidative stress [27]. The primary pharmacological interaction that should be considered is increased nonheme iron absorption, which may be positive in anemic patients but negative in iron overload disorders. VitC supplementation does not have any evidence of disrupting immunosuppressive or photodynamic therapy processes at standard doses [41,42].

Available data indicate that systemic VitC is generally safe, with good tolerability at doses of 1–2 g per day taken orally, and high-dose intravenous administration up to 50 g per day reported as safe when accompanied by appropriate renal surveillance [40,41,42,43,44]. Topical use is also usually well tolerated, although local irritation can occur, particularly on ulcerated mucosa, so buffered or pH-neutralized preparations are preferable [27]. Monitoring is recommended in patients with renal disease, a history of nephrolithiasis, or glucose-6-phosphate dehydrogenase (G6PD) deficiency [42,43]. Clinically relevant interactions appear limited, although increased iron absorption should be considered in individuals receiving concurrent iron supplementation.

Despite this favourable profile, there is a lack of OLP-specific safety and pharmacovigilance data, and adverse events together with renal parameters should be systematically collected in future randomized controlled trials [40,41,42,43,44]. Overall, systemic and topical VitC have a good safety and tolerability profile, and no significant drug interactions have been reported so far. The combination of these findings with its redox-modulatory capability warrants the use of VitC in controlled adjunctive trials of OLP.

## 7. Special Populations and Phenotypes

OLP is a chronic inflammatory disease of a heterogeneous nature. The oxidative-stress load, repair capacity of the epithelia, and the environment of salivary antioxidants vary between phenotypes and comorbid conditions. These aspects can affect the theoretical and practical application of VitC as a supplementary antioxidant treatment.

Xerostomia and hyposalivation lower lubrication, antimicrobial protection, and salivary antioxidant delivery to mucosal tissues, which is crucial in OLP, with redox imbalance worsening epithelial injury. Though it is not OLP-specific, a good clinical review by Villa et al. [47] offers comprehensive advice on how to evaluate and manage salivary-gland hypofunction. It also notes that the restoration of salivary flow and moisture promotes epithelial defence mechanisms; by proxy, local antioxidant delivery (e.g., VitC-enriched gels or rinses) may be a logical addition to OLP patients with xerostomia.

Tobacco smoking increases oxidative stress, inhibits immune response, and slows down mucosal healing. Although there is a paucity of direct OLP-specific evidence, these mechanisms suggest that smokers might need increased antioxidant supplementation and might not respond to supplementation clinically as effectively until cessation. Periodontal and mucosal studies have provided evidence to support the idea that chronic oxidative stress due to smoking decreases tissue VitC levels and impairs healing.

A few meta-analyses and mechanistic studies validate an increased oxidative-stress signature and disturbed antioxidant defences in OLP [9,12]. Studies in serum and saliva show mixed findings of VitC, with some reporting no difference to controls [24], and others showing a significant reduction in VitC in OLP patients [19]. Such differences are probably due to assay type, diet, and disease phenotype differences. Mechanistically, ROS play a role in the apoptosis of the keratinocytes, amplification of inflammatory cytokines, and slowing of wound repair, which offers a biologically plausible explanation of antioxidant interventions, such as VitC, to counteract the effects of ROS and facilitate wound healing [12].

Oral lichenoid lesions (OLL) and drug-induced reactions may resemble OLP histologically, but they are etiologically different and have different treatment priorities. Elimination of the precipitating drug or contact allergen is first line; antioxidant therapy can only be used as a supportive measure. Fortuna et al. [48] conducted a systematic review of medication-related lichenoid reactions and highlighted the inconsistency in causality determination, which is why a differentiation diagnosis is required prior to attributing the response, or non-response, to antioxidant treatment.

People with type 2 diabetes often show lower plasma vitamin C despite apparently adequate intake. Recent studies and cohort analyses indicate that diabetic patients have more frequent hypovitaminosis C, particularly with higher body weight or renal dysfunction, and may require about 1.4–1.6 times higher daily intakes, roughly ≥125 mg per day, to reach plasma levels comparable to non-diabetic individuals. Since both OLP and diabetes are associated with oxidative stress and, in some cohorts, reduced vitamin C status, diabetic OLP patients likely carry an additive deficit [23,49,50,51].

Diabetes trials and recent meta-analyses typically used 500–1000 mg per day of oral vitamin C for several weeks in addition to standard care and reported modest improvements in glycaemic control and some cardiovascular risk markers, with good short-term safety in patients without advanced nephropathy [52,53]. Extrapolating cautiously to OLP, it is reasonable in diabetic patients with normal renal function to ensure at least diet plus low dose supplementation to reach about 125 mg per day, and, if an adjunctive trial is considered, to use 500–1000 mg per day in divided doses for a limited period within usual upper intake limits. In patients with diabetic kidney disease, lower doses or food-based optimisation are preferable, and vitamin C should remain clearly an adjunct to established OLP and diabetes management, not a replacement.

Several important gaps and cautions must be considered when interpreting the current evidence. There are still relatively few trials that investigate VitC alone in OLP, and most available data come from studies using mixed antioxidant regimens rather than isolated VitC interventions [27]. Population-related modifiers such as xerostomia and smoking are likely to influence local VitC bioavailability and overall oxidative load, so these factors should be explicitly stratified in future clinical trials [47]. Methodological limitations, including heterogeneity of biomarkers, for example, serum versus saliva, and insufficient control of dietary intake, also make it difficult to define the true VitC status in OLP cohorts [19,24].

Against this background, research priorities include well-designed, subtype-stratified randomized controlled trials with standardized oxidative stress and clinical endpoints, to clarify the magnitude and specificity of any therapeutic benefit. Until such data are available, VitC should be regarded as an adjunctive measure, not a replacement for established anti-inflammatory interventions.

## 8. Conclusions

OLP is a complex, chronic, immune-mediated disease where oxidative stress is a precipitant and a potentiator of mucosal damage. The past ten years of evidence are consistent in showing high oxidative-damage indicators, low total antioxidant capacity, and loss of non-enzymatic antioxidants like VitC in the affected patients [9,10,15]. These biochemical imbalances are accompanied by epithelial vulnerability, slowed wound healing, and ongoing inflammatory stimulation, and so redox dysregulation plays a direct role in the maintenance of the disease [5,12].

VitC is a multifaceted biological agent that is consistent with these pathogenic processes. Being a major aqueous-phase antioxidant, it removes ROS and RNS, restores vitamin E, and promotes collagen hydroxylation and epithelial-barrier repair [13,16]. It also regulates the immune responses by inhibiting the expression of NF-kB and pro-inflammatory cytokines and enhancing the activity of regulatory T-cells [14]. These measures offer a viable mechanistic foundation of therapeutic value in OLP, especially in erosive or ulcerative phenotypes with high oxidative load.

The literature of biomarkers demonstrates that salivary VitC levels are often reduced in OLP compared to healthy controls, but serum results are less consistent, probably due to local intake and heterogeneity in methodology [10,15,19]. There is also evidence of clinical trials that contain VitC in antioxidant complexes like selenium-ACE or nano-vitamin preparations that show pain and lesion severity improvement with no serious adverse effects [26,27,28]. Even though no randomized controlled studies have been performed yet to investigate VitC monotherapy, the combination of biochemical and clinical evidence suggests that VitC monotherapy is a safe and reasonable addition to conventional anti-inflammatory treatment.

VitC is well tolerated and has a low drug-interaction risk at physiological to moderate supplemental doses (200–1000 mg/day) [13,42,43]. Topical delivery is a relatively new and promising route, as long as formulation stability and pH neutrality are attained [28,54]. Antioxidant support should be considered most beneficial to populations with xerostomia, smoking, diabetes, or nutritional deficiency, as they have an increased oxidative load [47].

Combined, existing data make VitC a biologically consistent, low-risk adjunct in the management of OLP and not a substitute for corticosteroids or calcineurin inhibitors. It must be included in future treatment regimens in rigorously designed, biomarker-based clinical trials with the capacity to establish dose–response relationships, the best delivery routes, and patient subgroups that are most likely to respond. Enhancement of this evidence base will be the key to whether VitC can become more than just a theory in OLP or become a standardized clinical use.

## Figures and Tables

**Table 1:** Summary of biomarker studies.

Compartment	Direction of Change in VitC	Associated Oxidative-Stress Pattern	Interpretation
Saliva	↓ VitC (consistent)	↑ MDA/TBARS, ↓ TAC, ↓ GSH	Local oxidative consumption and epithelial redox stress
Serum	Mixed (↓ in 1 study, no change in 1)	↑ MDA/8-isoprostane, ↓ TAC	Possible systemic utilization and inflammatory depletion
Panels	VitC ↓ correlates with TAC ↓ and MDA ↑	Broad oxidative imbalance across tissues	Supports the integrated oxidative-consumption model

Notes. ↑—increase, ↓—decrease.

## Data Availability

No new data were created or analyzed in this study. Data sharing is not applicable to this article.

## Abbreviations

The following abbreviations are used in this manuscript:

GPx	Glutathione peroxidase
GSH	Glutathione
IFN	Interferon
IL	Interleukin
MAPK	Mitogen-activated protein kinase
MDA	Malondialdehyde
MMP	Matrix metalloproteinases
NF-κB	Nuclear factor kappa-light-chain-enhancer of activated B cells
OLP	Oral lichen planus
PDT	Photodynamic therapy
RNS	Reactive nitrogen species
ROS	Reactive oxygen species
SOD	Superoxide dismutase
SVCT1/2	Sodium-dependent vitamin C transporter 1/2
TAC	Total antioxidant capacity
TAL	Total antioxidant levels
TBARS	Thiobarbituric acid reactive substances
Th1/Th2/Th17	T helper cell type 1/2/17
TNF-α	Tumour necrosis factor alpha
